# Consequences of Non-Intervention for Infectious Disease in African Great Apes

**DOI:** 10.1371/journal.pone.0029030

**Published:** 2011-12-22

**Authors:** Sadie J. Ryan, Peter D. Walsh

**Affiliations:** 1 Department of Environmental and Forest Biology, SUNY College of Environmental Science and Forestry, Syracuse, New York, United States of America; 2 Department of Archaeology and Anthropology, University of Cambridge, Cambridge, United Kingdom; Texas A&M University, United States of America

## Abstract

Infectious disease has recently joined poaching and habitat loss as a major threat to African apes. Both “naturally” occurring pathogens, such as Ebola and Simian Immunodeficiency Virus (SIV), and respiratory pathogens transmitted from humans, have been confirmed as important sources of mortality in wild gorillas and chimpanzees. While awareness of the threat has increased, interventions such as vaccination and treatment remain controversial. Here we explore both the risk of disease to African apes, and the status of potential responses. Through synthesis of published data, we summarize prior disease impact on African apes. We then use a simple demographic model to illustrate the resilience of a well-known gorilla population to disease, modeled on prior documented outbreaks. We found that the predicted recovery time for this specific gorilla population from a **single** outbreak ranged from 5 years for a low mortality (4%) respiratory outbreak, to 131 years for an Ebola outbreak that killed 96% of the population. This shows that mortality rates comparable to those recently reported for disease outbreaks in wild populations are not sustainable. This is particularly troubling given the rising pathogen risk created by increasing habituation of wild apes for tourism, and the growth of human populations surrounding protected areas. We assess potential future disease spillover risk in terms of vaccination rates amongst humans that may come into contact with wild apes, and the availability of vaccines against potentially threatening diseases. We discuss and evaluate non-interventionist responses such as limiting tourist access to apes, community health programs, and safety, logistic, and cost issues that constrain the potential of vaccination.

## Introduction

Poaching and habitat loss are known major threats to African apes [1], but what has only in the last few years come into focus is that infectious disease is a threat of similar magnitude. Wild populations of gorillas and chimpanzees are threatened by a diverse array of virulent pathogens, including Ebola virus [1], [2], Anthrax [3], simian immunodeficieny virus (SIV) [4], and a variety of human respiratory viruses [5], [6]. These recent illustrations of the magnitude of disease threat have intensified a longstanding debate about the advisability of medical interventions such as vaccination. The dissent seems to hinges on two major points, one on the ethics of intervening in “natural” systems and the other on the magnitude of threat and cost effectiveness of vaccination relative to other conservation strategies.

Our goal is to explore the potential impact of disease outbreaks on African great apes, and the available interventions, such as vaccination and treatment, as practical conservation strategies. More precisely, our objective is to provide a scientifically based discussion about the need for, feasibility and cost effectiveness of intervention for disease threats in African apes.

1. We present an overview of previous studies to review pathogens known to have infected wild African apes, and describe the population impact they are known to have had, to allow the reader to gauge the magnitude of the disease threat. 2. We parameterize a simple demographic model to project the time scales over which a well-known gorilla population would recover from outbreaks of known previous diseases, to illustrate how little resilience ape populations have to disease. 3. We then assess future disease risk, in terms of the prevalence of several potentially dangerous pathogens in human populations and the rates of vaccination against them, both in African ape range states and in a typical source country for ape tourism programs. 4. We synthesize the available literature and reports on current vaccine status for both apes and humans, for diseases known to impact great apes. We then discuss and compare approaches to mitigating disease impact on wild apes, from behavior guidelines for tourist and staff to local human community health programs to ape health intervention measures. We then focus our discussion on efforts to treat wild apes for disease and compare these to the potential vaccination has for protecting wild apes against disease, including the status of available human vaccines. We address the practicalities of vaccination, including safety, cost, and vaccine delivery, and close with some thoughts on the ethics of vaccination and other medical interventions.

### Known disease threats to African apes

Pathogens that threaten wild gorillas and chimpanzees fall into three broad classes, pathogens that circulate persistently in other forest animals (sylvatic pathogens) then occasionally spill over into apes, pathogens that spillover from humans (reverse zoonotic pathogens), and pathogens that circulate persistently within wild ape populations (enzootic pathogens). Perhaps the best known pathogen to recently threaten African apes is the Ebola Virus. Over the last two decades the Zaire strain of Ebola has killed roughly one third of the world's gorilla population and only a slightly smaller proportion of the world's chimpanzees [1], [2], [7]. Although these large Ebola Zaire outbreaks in great apes have been documented only in Gabon and the Republic of Congo [8], [9], chimpanzees in Ivory Coast have been killed by another strain, Ebola Cote d'Ivoire [10].

Human filovirus outbreaks have also occurred in several other African ape range states, including Angola (Marburg virus) [11], Democratic Republic of Congo (Ebola Zaire and Marburg virus) [12], [13], and Uganda (Marburg Virus, Ebola Sudan and the newly discovered Bundibugyo strain of Ebola) [14], [15], [16]. The 2007 human outbreaks of Ebola Bundibugyo occurred close to several chimpanzee populations: Semliki National Park (12 km away), Rwenzori National Park (36 km away), Toro (Semliki) Game Reserve (41 km), and Kibale National Park (47 km away), and Marburg virus was detected in bats living at Kitaka Caves, 65 km south of Kibale [17].

The first evidence that enzootic diseases also pose a threat to wild apes was also reported in 2009. A combination of clinical observations, demographic analyses, and pathogen assays showed that simian immunodeficiency virus (SIV) is not non-virulent in chimpanzees, as previously suggested by captive studies. Rather, wild, SIV infected chimpanzees showed AIDS-like symptoms, birth rates about one third of uninfected animals, and annual mortality rates about ten times higher [4]. These observations raise the concern that other pathogens known to persistently infect wild apes, such as simian foamy viruses [18], hepatitis B [19] regularly circulating adenoviruses [20] and malaria [21] may also negatively influence birth or survival rates.

It is increasingly clear that a number of pathogens spilling over from humans represent a severe threat. It has been documented for at least a decade that the growth of human populations surrounding parks in east Africa has resulted in transmission of human gastrointestinal parasites to wild apes [22], [23]. However it is only in the last three years that modern molecular methods have confirmed longtime fears that habituating wild gorillas and chimpanzees to human presence would increase rates of human respiratory pathogen transmission. Not only do phylogenetic analyses show a close affinity between globally circulating human viruses and respiratory viruses that killed chimpanzees at two sites and gorillas at a third [24], [25], [26], but clinical observations suggest that respiratory disease may have been a major source of mortality at four of the longest studied chimpanzee habituation sites [5], [24], [27], [28]. At a recent workshop on great ape health, representatives from virtually all gorilla and chimpanzee habituation sites in attendance reported observing clinical symptoms consistent with respiratory disease (Symposium on Great Ape Health, Kampala, Uganda 2009).

The importance of infectious disease as a threat to wild apes should be measured not just in terms of the number of deaths caused by disease outbreaks but also in terms of ape population resilience: the time necessary for a population to recover from disease mortalities. Population resilience is central to assessing the disease threat because gorillas and chimpanzees reproduce more slowly than virtually any other animal on earth, including humans.

## Materials and Methods

To characterize mortality rates typical of disease outbreaks in wild apes we compiled data from sixteen previously published outbreaks, wherein community size, number infected and the mortality rate were explicitly reported (Table 1). While the etiological agent was not always confirmed with laboratory diagnostics, the class of disease (e.g. respiratory infection versus hemorrhagic fever) was. The impact of disease from a single outbreak ranged widely: e.g. respiratory outbreaks had 0–25% mortality while Ebola Zaire outbreaks exhibited mortality rates of 95% or greater.

**Table 1:** Sixteen previous disease outbreaks in African great apes, for which published estimates of mortality are available.

*Disease outbreak*	*α(Reference)*
Respiratory (Gombe, 2000)	4 [24]
Mange (Gombe, 1997)	6 [24]
Respiratory (Gombe, 1968)	8 [24]
Polio (Gombe, 1966)	10 [24]
Flu-like (Mahale, September 1993)	10.8 [58]
Mystery (Tai, 1993)	10.8 [25]
Ebola Cote d'Ivoire (Tai, 1994)	12.2 [25]
Flu-like (Mahale, December 1994)	14.8 [58]
Respiratory (Gombe, 1987)	17 [24]
Flu-like (Mahale, 2006)	18.5 [28]
Flu-like (Bossou, 2003)	25 [59], [60]
STLV or Strep (Tai, 1999)	31.25 [25]
Ebola, (Lossi Chimpanzees)	77 [2]
Ebola (Lossi Gorillas, 2002–2003)	91 [2]
Ebola (Lossi Gorillas, 2003–2004)	96 [2]

Summary of sixteen previously published outbreaks for which the mortality impact, the percentage mortality in the group, α, is given, or was possible to estimate. Note that, for many of the outbreaks, the pathogen was not explicitly identified.

To demonstrate the resilience of populations to disease outbreaks, we used a demographic modeling exercise.

To describe population growth in gorillas we used a discrete, logistic model:
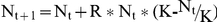
We parameterized the model using an estimated demographic rate (*R*) for gorillas in the Virunga Mountains of Rwanda. To be conservative, we used highly optimistic estimates which tended to overestimate gorilla reproductive potential. They yielded a Leslie matrix estimate for the annual rate of increase of *R* = 3.7% (Walsh and Caillaud, *unpublished*). Using gorillas was conservative in that chimpanzees have an even lower maximum population growth rate, probably about 2% per year.

For the population size before disease impact (*N*), and the carrying capacity (*K*), we used estimates from the 1997 Population and Habitat Viability Analysis (PHVA) for gorillas in Uganda's Bwindi National Park [29]. The estimated population size was 320 gorillas, and the carrying capacity estimate (*K*) was 300–500, so we used a midpoint of *K* = 400.

**Figure 1:**
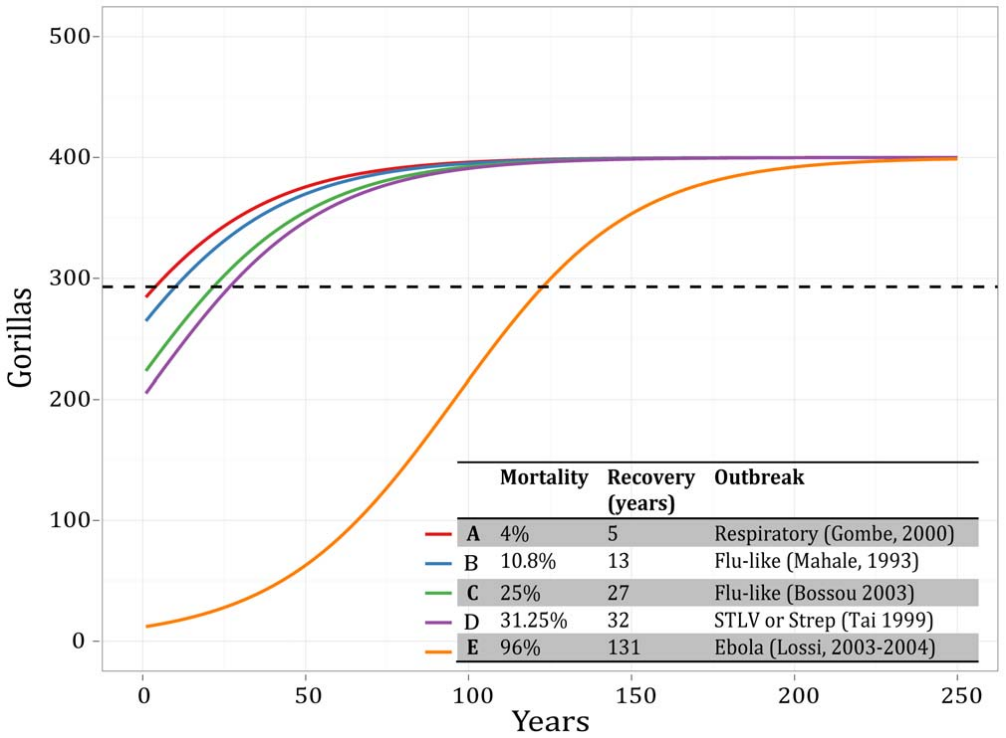
Recovery curves for hypothetical outbreak scenarios within a gorilla population. Five outbreaks from Table 1, (Respiratory (Gombe, 2000), Flu-like (Mahale, 1993), Flu-like (Bossou 2003), STLV or Strep (Tai 1999), Ebola (Lossi, 2003–2004)) are used to demonstrate the recovery time to initial population size (dashed line), for a given outbreak mortality.

We considered a series of five scenarios in which proportional mortality rate, α, corresponded to the mortality rate observed in a real outbreak. In each scenario, we seeded the logistic growth model with an initial, post-outbreak population size of
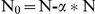
then iterated the logistic model in annual times steps until gorilla population size reached the initial population size, *N*, as a measure of recovery time. To examine the resilience of gorillas to disease we considered five disease mortality scenarios that spanned the mortality rates reported in Table 1, and the resulting trajectories are shown in Figure 1.

To assess future potential spillover disease risk, we examined human vaccination rates and reported cases (where available) for five exemplar great ape range countries using the UNICEF/WHO 2009 global immunization summary [30] and the WHO 2009 WHS (World Health Statistics) [31], for seven diseases known to be communicable to great apes (Table 2). We also included data from the United Kingdom, a leading source country for ape tourists.

**Table 2:** Vaccination coverage (%) and (cases) reported by country (not reported denoted nr).

*Disease (Vaccine)/Country*	*Measles (MCV)*	*Mumps*	*Rubella*	*Pertussis (DPT1/DPT3)*	*Polio (Pol3)*	*Tetanus (TT2)*	*TB (BCG)*
**Congo**	67 (84)	0	nr (2)	80/80 (55)	80 (0)	90 (3)	86 (3,552)
**Cote d'Ivoire**	67 (5)	nr	nr (48)	93/76 (nr)	75 (0)	76 (31)	94 (14,071)
**DRC**	79 (55,577)	nr	nr	95/87 (3,799)	87 (41)	81 (1,153)	94 (66,099)
**CAR**	62 (49)	nr (0)	nr (118)	65/54 (2)	47 (0)	54 (68)	74 (nr)
**Gabon**	55 (0)	nr	nr (0)	69/38 (nr)	31 (0)	67 (nr)	89 (1,462)
**Uganda**	68 (3,776)	nr	nr (605)	90/64 (nr)	59 (0)	85 (1,007)	90 (21,303)
**UK**	86 (1,022)	nr (2 569)	nr (31)	97/92 (1,163)	92 (0)	nr (4)	75^a^ (1,639)

a Coverage reported in 2005 European survey [61], as not reported in WHO statistics.

Cases reported for human diseases and vaccination coverage (%) in exemplar great ape range countries (Congo, Cote d'Ivore, Democratic Republic of Congo (DRC), Central African Republic (CAR), Gabon and Uganda) and a tourist country (United Kingdom (UK)). MCV is a Measles Containing Vaccine, including MMR (Mumps Measles Rubella); DPT1/DPT3 are the first and third Diptheria, Pertussis and Tetanus vaccinations given, so the rate at which the third is given is likely best representation of coverage. Similarly for the Polio vaccine, Pol3 is the third in a series given. For Tetanus, the TT2 is the second of five in a series, and TT2 confers up to 5 years of expected protection, and is usually given to pregnant mothers to prevent neonatal tetanus. The tuberculosis (TB) vaccine, the Bacillus Calmette-Guérin (BCG) attenuated bovine tuberculosis strain, is thought to be around 80% effective for 15 years, but this is highly dependent on geography and presence of strain types.

We conducted a literature review of human vaccines for pathogens that were either already known to infect wild apes or presented a high risk of infection (e.g. respiratory pathogens likely to be carried by tourists). For each pathogen we scored whether at least one vaccine was licensed (L) or under development; in the advanced stage of development (A) if the most advanced vaccine was in human clinical trials; or in the early stage of development (E), if the most advanced vaccine was not yet in human clinical trials but had protected captive non-human primates from pathogen challenge. We also identified mode of transmission, the identity of the reservoir host, and the likely duration of vaccine-induced immunity (Table 3).

**Table 3:** Vaccinations available for wild ape populations.

*Pathogen*	*T*	*WA*	*R*	*ID*	*LS*
Measles Virus	R	*	Human	L	L [62]
Mumps Virus	R		Human	L	L [62]
Rubella Virus	R		Human	L	L [62]
Influenza Virus	R		Human	S	L [62]
Varicella Virus (chickenpox)	R		Human	L	L [62]
Respiratory syncitial Virus (RSV)	R	*	Human	S	E [63]
Human Metapneumovirus	R	*	Human	S	E [64]
Diptheria Virus	R		Human	L	L [62]
Pertussis Virus (whooping cough)	R		Human	L	L [62]
*Streptoccocus pneumoniae*	R	*	Human	S	L [62]
Hepatitis A Virus	F		Human	L	L [62]
Hepatitis B Virus	S	*	Ape	L	L [62]
Tuberculosis	R		Human	L	L [62]
Polio Virus	F	*	Human	L	L [62]
Rabies Virus	B		Domestic Dog	S	L [62]
Ebola Virus	?	*	Bat?	U	A [65], [66], [67]
Anthrax	?	*	?	L	L [62]
Malaria	V	*	Ape	U	A* [68], [69]
Tetanus	W		?	S	L [62]
Simian Immunodificiency Virus (SIV)	B,S?	*	Ape	U	E* [70]
Dengue Fever Virus	V		Primate	U	E [71]
Yellow Fever Virus	V		Primate	L	L [62]

Vaccinations available for wild ape populations, by **(T)** **transmission** mode (R = Respiratory, B = Bite, F = Fecal, V = Vectorborne, W = Wound, S = Sexual), whether the pathogen has been explicitly identified in **(WA)** **wild apes** (*), the disease **(R)** **reservoir**, the **(ID)** **immunity duration** (Longterm (L), Shortterm (S) or Unknown (U)) of the vaccination, and **(LS)** **licensing status**, as described in the methods (L = Licensed, E = Early experimental, A = Advanced experimental, * = Vaccine for human strain only).

## Results

We found that the predicted recovery time for this specific gorilla population from a ***single*** disease outbreak ranged from 5 years for a low mortality (4%) respiratory disease outbreak, to 131 years for an Ebola outbreak that killed 96% of the gorilla population. Even this bleak picture of resilience for a well-known gorilla population is highly optimistic. The demographic rates, the simplicity of recovery, and the assumption of only a single outbreak event are ‘conservative’ treatments of the potential problem. In particular, our approach assumes no further disease introduction or spread after the initial outbreak and that a population of gorillas will not collapse at low numbers (no Allee effects), but rebound and continue stable growth. In addition, in this model, we used a purely deterministic framework for illustrative purposes. For populations this small, stochastic population models are likely more appropriate to understand the potential impact of diseases, particularly in the context of demographic fluctuations. Recovery of the population from such a severe impact as our Ebola outbreak simulation is questionable, meaning that this is a highly conservative illustration, and must be seen as such.

However, in great apes habituated for tourism, we can expect frequent introductions of human pathogens. In the case of rapidly evolving viruses such as influenza and respiratory syncytial virus (RSV) this is likely to involve multiple distinct strains with little or no cross-immunity. RSV and human metapneumovirus (hMPV), are seasonally cycling human respiratory infections, like influenza. They are often unidentified, due to symptomatic similarity to the common cold. In the developing world, they also constitute a major source of infant mortality [32], [33], have been confirmed as sources of substantial chimpanzee mortality at two sites [5], [6], and may well be responsible for the many of the unidentified “flu-like” outbreaks described in Table 1.

### Predicting future spillover risk

There is little doubt that the rate of pathogen spillover from humans to African great apes is increasing. One major reason is that the lure of tourist revenue is leading national governments to habituate more ape social groups for tourism. Because of the scarcity of diagnostic data on exactly which pathogens infect apes and at what rates it is difficult to rigorously quantify how increased tourism will translate into increased disease pressure on ape populations. However, it is possible to quantify the pool of pathogens that apes in tourism programs are exposed to in terms of the disease load in both tourists and the national staff that work in habituation programs. We assessed this disease load in terms of human vaccination rates and reported cases (where available) for five exemplar great ape range countries using the UNICEF/WHO 2009 global immunization summary [30] and the WHO 2009 WHS (World Health Statistics) [31], for seven diseases known to be communicable to great apes (Table 2). We also included data from the United Kingdom, a leading source country for ape tourists.

On one hand, the prevalence data are encouraging, as they do not reflect the extremely high childhood respiratory disease (measles, mumps, rubella, pertussis) rates that until recently characterized equatorial Africa. This is largely due to a massive push over the last decade to improve vaccination rates [34], with large-scale international efforts such as the Measles Initiative (the Measles Initiative: the American Red Cross, UNICEF, the United Nations Foundation, the United States Centers for Disease Control and Prevention (CDC) and the World Health Organization (WHO)). On the other hand, these data are discouraging, in the sense that despite a massive infusion of funds, vaccination rates for target pathogens such as measles have only reached the mid-60% range (Table 2), and these pathogens continue to circulate in the region. Measles still kills about 200,000 children each year, despite a 91% reduction in cases in Africa reported between 2000 and 2006 [35]. This is a cause for concern in that any failure to sustain funding (particularly problematic in Africa) could result in a return of the regional massive outbreaks that were characteristic prior to 2000. Furthermore, the prevalence of circulating acute respiratory pathogens (e.g. RSV, HMPV) that are not currently vaccine treatable, is increasing, due to rising rates of international air travel [36], [37]. Chronic diseases such as tuberculosis also remain a problem in Africa, and Polio has recently made a resurgence [38]. Moreover, even a developed country such as the UK shows only a 86% measles vaccination rate; possibly as a consequence of fears about vaccine safety that have now shown to be unfounded [39].

The local “background” spillover rate for fecal-oral pathogens is also increasing due to the combined effects of increased human population densities around parks, encroachment into protected areas, habitat degradation, and even habituation, which forces apes out of protected areas in search of food [23], [40]. Protected areas in East and West Africa are already small islandized parks in highly populated areas [41], [42], [43], and Central African protected areas are destined to follow in coming decades.

## Discussion

Infectious disease is a serious a threat to African apes, along with poaching and habitat loss. This threat is likely to increase as human disease spillover into wild ape populations intensifies, both because of rising population pressure around protected areas and because of increasing ape tourism.

### Alternatives for Disease Mitigation

We hope that our overview of past disease impact, population resilience, and future disease risk illustrates convincingly that infectious disease is a serious problem for African great apes that requires a response. The options for this response vary from “hands off” approaches such as educating governments about the costs of too much tourism, stricter enforcement of health guidelines for approaching habituated animals, stricter exclusion of humans from protected areas, and health programs for staff and local populations, to more interventionist approaches such as treatment and vaccination of gorillas or chimpanzees. In the following paragraphs, we attempt to highlight two issues. First, that the appropriate response(s) depends upon the source of infection, and second, that cost-effectiveness should be a major consideration in choosing responses to a given threat.

### Optimal rates of tourism

One option for blocking the spillover of human respiratory viruses might be to entirely stop habituation of gorillas and chimpanzees for tourism. However, great ape tourism is a substantial source of revenue for national governments, park budgets, politically powerful tour operators, and local communities. Consequently, an outright ban on tourism would not only be politically impractical but would likely result in the deterioration of both protective efforts by park management authorities and compliance with park regulations by local communities. The increased impact of other threats such as poaching and habitat degradation would then likely outweigh any benefits of disease control.

A more promising middle path is to educate local stakeholders on the fact that tourism revenue is not maximized by maximizing the number of tourists that visit. It may be useful to view this as a maximum sustainable yield problem in which harvesting is replaced with disease impact. Increasing the tourism rate is like increasing harvest rate, it increases short term revenue but it also increases the rate of disease introduction and, therefore, reduces the population growth rate of the exploited species. In other words, increasing the rate of tourism eventually decreases the number of gorillas or chimpanzees available for viewing by tourists and, therefore, decreases tourism revenue. In the long term, tourism revenue is actually maximized by not bringing too many people.

There are two impediments to exploiting the maximum sustainable yield concept. The first is data scarcity. Choosing an optimal visitation rate requires information about the relationship between tourist visitation rates and rates of great ape mortality or reproductive impairment. Long term demographic data are already available at some sites [24], [44], but vigorous efforts need to be made to improve data collection at other sites, with a particular emphasis on quantifying the response of birth and death rates to fluctuations in the rate of tourism. The second impediment is that the maximum sustainable yield concept is admittedly somewhat counterintuitive. Consequently, convincing local stakeholders that restraint is in their long term self-interest will like require substantial creativity and persistence.

### Hygiene and behavior guidelines

A complementary alternative to limiting the number of tourists is to limit the exposure risk posed by each tourist. Current Best Practice guidelines [45] include a variety of strategies for limiting disease spillover, including wearing facemasks, observing minimum approach distances, limiting visit duration, prohibiting discharge of body fluids (e.g. spitting, defecating) in the forest, and barring tourists or staff that exhibit symptoms of infection (e.g. coughing, runny nose, fever) or do not present vaccination records [46].

While these are well thought out and useful guidelines, there are again two major obstacles. Firstly, there are currently no published data on the efficacy of these measures in preventing disease spillover. For example, studies estimating the distance necessary to prevent respiratory virus spillover, how long a visit can last, and whether masks need to be worn only when in close proximity to apes or at all times, simply do not exist, so current guidelines [45] are based on “best guesses”. This is important because although the precautionary principle advises making the guidelines as stringent as possible, the economic imperative pushes in the opposite direction. Having only a short visit, wearing a hot and sticky mask, standing far away from the animals, and not being able to spit or urinate for several hours degrades the tourism experience and, presumably, both the number of tourists who want to come and the amount they are willing to pay. Thus, we are again balancing disease exposure risk with tourism revenue, which ultimately determines the intensity of other conservation threats. Quantifying how different rules affect spillover rates is necessary so that guidelines can be set in a way.

The second, more serious problem, is compliance. Park authorities and tourist guides have strong economic incentives to let tourists go without masks, approach too close, stay too long, and visit when they are ill: both for promoting future tourism and for obtaining tips. Consequently, strict safety guidelines are not enforced at most ape tourism sites in Africa [47], [48], [49]. Thus, although hygiene and behavior guidelines have great potential, and should be vigorously pursued, in practice we cannot currently rely on them as the sole method for protecting habituated chimpanzees and gorillas from diseases carried by tourists and tourism personnel.

### Exclusion of humans from protected areas

When humans enter protected areas to engage in activities such as hunting or wood gathering, they may leave potentially infectious fluids that can infect wild chimpanzees and gorillas [22], [23]. Human feces are a particular problem because fecal micro and macro-parasites are typically more resistant to environmental degradation than are other parasites (e.g. respiratory viruses). Policing the defecatory habits of people who are already in the forest illegally is exceptionally difficult. Rather, a more logistically feasible approach is simply to prohibit all unauthorized entry into protected areas for apes. In principle, this is already the policy at most protected areas for African apes. However, in practice, enforcement is often weak, as evidenced by high rates of hunting and habitat degradation at many great ape protected areas. This approach would therefore require an increase in both personnel to guard park borders, and mechanisms for enforcement.

### Employee and community health programs

Another option for limiting disease spillover is the establishment of health programs for staff involved in the habituation of apes for tourism or research, including vaccination, screening and treatment. This approach has the advantage of both blocking disease spillover and enhancing employee loyalty. However, it also entails ethical and economic subtleties that need to be weighed carefully, such as whether to screen for diseases for which treatment is unaffordable, and whether treatment for diseases that are particularly communicable to gorillas and chimpanzees should receive priority over chronic or non-infectious diseases that are not. These ethical questions add complexity to what might appear to be a simple solution.

A further option is the extension of health programs to local communities surrounding the protected areas in which apes live. For example, infection of wild apes in Uganda by gastrointestinal parasites and pathogens appears to occur not just by movement of humans into protected areas, or of wild apes out, but also through waterborne transport from upstream villages [22]. This problem might be addressed by health interventions in local human populations, such as medication, installation of water filtration systems and education programs directed at water use and hygiene. However, we are again faced with economic and ethical questions: which is more sustainable, an ape treatment program that could be undertaken by a single park-based veterinarian, or a much larger public health effort? Community health programs may have the added benefit of strengthening bonds with local populations, and adding support to ape conservation programs. But it is important not to confuse political objectives with the actual control of disease spillover when community support may be achieved more cost-effectively. This is particularly important at the conservation – public health interface, where funding may be directed by larger agencies with formal missions and objectives that may not align well with conservation goals.

### Treatment

Curative treatment – that is, reactive intervention - is rare or absent at most ape conservation sites, but plays a regular role in management in the tiny remnant populations of mountain gorillas [46], [50]. Infection associated with injury is a common cause for treatment. Gorillas are also periodically treated for parasites such as sarcoptic mange [51]. Intervention in the form of treatment may be the most cost-effective means of controlling fecally transmitted parasites, particularly if efficient methods of oral drug delivery can be developed (which would minimize safety concerns associated with darting).

Unfortunately, treatment is currently not a promising measure for acute outbreaks of respiratory and hemorrhagic viruses. For instance, there are no licensed anti-viral drugs effective against hemorrhagic viruses such as Ebola virus, at present. Current anti-viral drugs also show limited effectiveness against respiratory viruses, although new, more effective anti-virals are under development [52], [53], and antibiotics can be effective against secondary bacterial infections. Importantly, the veterinary infrastructure necessary to effectively implement treatment for great apes in response to acute outbreaks is substantial. Because respiratory pathogens spread rapidly through ape communities [5] and because anti-virals and antibiotics are most effective when administered early in infection [54], effective control of acute outbreaks through treatment likely requires both the permanent presence of veterinary personnel on site and storage of large numbers of treatment doses. It also presupposes a well-maintained monitoring system for quickly detecting symptoms and that large numbers of animals can be quickly, safely, and (perhaps) repeatedly treated – presumably using hypodermic darts. On site diagnostic capacity is also particularly important, as inappropriate treatment (e.g. antiviral drugs for bacterial infection) must be minimized. This is essential to reduce the risk of evolved drug resistance, particularly in a context where it may be difficult to ensure that treatment regimes are completed.

### Vaccination

To our knowledge, wild apes have been the object of population-wide vaccination campaigns on only two occasions: emergency vaccination efforts to protect chimpanzees from a presumed polio outbreak at Gombe, Tanzania [7] and vaccination of gorillas during a measles outbreak in the Virungas [55]. A handful of mountain gorillas have also been vaccinated against tetanus on an opportunistic basis when immobilized for treatment of snare wounds (*C. Whittier, pers. comm.*).

Our review of available vaccines suggests that there are currently a large number of human vaccines that might be used to protect wild apes (Table 3). We found vaccines for twenty-two different pathogens that are known to, or could potentially threaten wild apes, of which sixteen vaccines are already licensed. We emphasize that this is not an exhaustive list, merely our best guess at which vaccine preventable diseases were of greatest threat to wild apes. In our findings, vaccines for respiratory pathogens were the most common, and humans were the most common reservoir host.

One roadblock to using these vaccines as conservation tools is simply getting the vaccine into wild gorillas and chimpanzees. In the long term, the most desirable means of vaccine delivery is oral: that is packaging the vaccine in a bait that is eaten by gorillas and chimpanzees. However, oral baiting involves a series of technical, financial and political challenges that limits its near term potential. For instance, although using a natural fruit as a bait might seem ideal, the acids in the fruit can rapidly degrade the vaccine. In order to avoid transmission of other human pathogens, baits also need to be packaged under sterile conditions, which is difficult in the field. Thus, an artificial, manufactured bait may be the best solution, particularly if large numbers of baits are to be distributed (e.g. to unhabituated animals). Both finding an artificial bait that wild apes will eat, and formulating vaccines in heat stable, environmentally robust forms that can be packaged in baits, are non-trivial technical tasks.

Baiting also introduces additional safety concerns, as vaccines that are most effective for oral delivery are typically replicating. That is, they are infectious agents in which viral reproduction has been attenuated but are still capable of causing a mild infection in the target animal. One fear is that under uncontrolled field conditions and in immunologically stressed wild animals, such vaccines could cause severe infections or mutate to more virulent forms. This risk is magnified when the baits may be consumed by non-target species in which the vaccine has not been studied. This, in turn, necessitates higher standards of safety testing than that for vaccines delivered through other means (e.g. by hypodermic dart) and thus raises the costs of oral vaccination. The cost of baiting is also increased because a large number of vaccine doses (e.g. 100–1,000) might need to be distributed for every dose actually consumed by an ape.

Having duly mentioned these safety and cost concerns, we think that with careful attention they can be overcome. For instance, the deployment of hundreds of millions of oral baits led to the virtual eradication of fox rabies in Europe with almost no recorded spillover into humans [56]. Likewise, oral vaccination seems the best option in the long run because it might allow the vaccination of a large number of unhabituated apes against spillover pathogens such as Ebola and (in the future) enzootic pathogens such as SIV, as well as repeated vaccination of habituated apes against rapidly evolving human respiratory pathogens.

In the meantime, the best way forward seems to be vaccine delivery using a hypodermic dart. Darting is not without problems, most prominently the risk of injury to darter and dartee. But several decades of darting mountain gorillas [46], [57] suggest that these risks can be minimized, particularly because vaccination of gorillas and chimpanzees does not require immobilization. Taking an incremental approach with darting would allow us to develop the epidemiological assays and field protocols to insure that wild apes can be vaccinated safely and effectively before moving to the more complex challenges of oral vaccination.

Some readers may object to vaccination on the grounds that the conservation objective should be to maintain the “natural balance”. Consequently, we should only be concerned with diseases introduced by humans. However, modern human activities are now upsetting the “natural balance” in Equatorial Africa in massive and unprecedented ways. The extraction of timber, oil, and minerals for export to developed countries is destroying vast tracts of habitat. The jobs created by these export industries, and the food and medicines imported from developed countries have allowed local human populations to explode to many times their historic levels, creating unprecedented demand for agricultural land and firewood as well as a cash market for bushmeat. Ecological communities and ecosystems are so affected by local, regional and global level anthropogenic impact that we suggest that it is no longer clear what “natural” means. Thus, even for pathogens such as Ebola, SIV, or malaria, which are originally enzootic, we now likely need to intervene in “natural” diseases that handicap the resilience of wild ape populations to other threats.

On a more practical level, direct health interventions for great apes could be highly cost-effective. For example, treatment of the relatively small number (at most hundreds) of gorillas or chimpanzees in a park that is heavily affected by fecal pathogen spillover would be much less expensive than health programs directed at thousands or tens of thousands of people living adjacent to the park. Although perceptions of the costs of vaccination are dominated by the tens of millions of dollars invested in developing vaccines for the human market, the per-dose price of many licensed vaccines is very modest: often only a few dollars. Vaccination is likely cost-effective as it would not require as much veterinary infrastructure as is necessary for treatment, and can be conducted under non-emergency conditions. In addition, vaccination would not require such a high level of sustained disease surveillance or the on-site maintenance of permanent veterinary teams, diagnostic capacity, or large standing stocks of drugs. In fact, a single roving vaccination team might cover many great ape sites. These low overhead costs thus give vaccination a high potential for sustainability, once vaccines are made available.

Tourism provides a substantial amount of the revenue for conservation of African great ape populations. Thus it is very hard to limit this route of disease spillover to great apes. To some extent, the disease threat to African apes could be diminished through non-interventionist approaches such as limitations on tourist numbers and behavior or staff and community health programs. However, non-interventionist approaches alone seem unlikely to entirely contain the disease threat. To be effective, limits on tourist numbers and behavior must be rigorously enforced; unfortunately, enforcement is notoriously lax at ape tourism sites. This problem is compounded by the fact that tactics aimed at preventing disease spillover from tourists tend to conflict directly with the profit motive. Additionally, programs focused on preventing human disease spillover do not address the threat posed by non-human diseases (e.g. Ebola, malaria, SIV, or hepatitis B), which have a major impact on ape population growth rates.

Based on our research here, we suggest that the great ape conservation community should pursue and promote treatment and vaccination, as weapons in the arsenal for fighting the decline of African apes. This should include rigorous assessments of both safety and cost-effectiveness, and should emphasize program sustainability, with particular attention to the training of African veterinary personnel. Field studies on safe and efficient methods for delivering treatments and vaccines orally should be a priority, but there is also a critical need for studies evaluating the cost-effectiveness of all ape conservation strategies in terms of their marginal effects on ape viability.

## References

[1] Walsh PD, Abernethy KA, Bermejo M, Beyersk R, De Wachter P (2003). Catastrophic ape decline in western equatorial Africa.. Nature.

[2] Bermejo M, Rodriguez-Teijeiro JD, Illera G, Barroso A, Vila C (2006). Ebola Outbreak Killed 5000 Gorillas.. Science.

[3] Leendertz F, Lankester F, Guislain P, Néel C, Drori O (2006). Anthrax in Western and Central African great apes.. American Journal of Primatology.

[4] Keele BF, Jones JH, Terio KA, Estes JD, Rudicell RS (2009). Increased mortality and AIDS-like immunopathology in wild chimpanzees infected with SIVcpz.. Nature.

[5] Kondgen S, Kuhl H, N'Goran PK, Walsh PD, Schenk S (2008). Pandemic human viruses cause decline of endangered great apes.. Current Biology.

[6] Kaur T, Singh J, Tong S, Humphrey C, Clevenger D (2008). Descriptive epidemiology of fatal respiratory outbreaks and detection of a human-related metapneumovirus in wild chimpanzees (*Pan troglodytes*) at Mahale Mountains National Park, Western Tanzania American. Journal of Primatology.

[7] Woodford MH, Butynski TM, Karesh WB (2002). Habituating the great apes: the disease risks.. Oryx.

[8] Rouquet P, Froment JM, Bermejo M, Kilbourn A, Karesh W (2005). Wild animal mortality monitoring and human Ebola outbreaks, Gabon and Republic of Congo, 2001–2003.. Emerging Infectious Diseases.

[9] Caillaud D, Levréro F, Cristescu R, Gatti S, Dewas M (2006). Gorilla susceptibility to Ebola virus: The cost of sociality.. Current Biology.

[10] Le Guenno B, Formenty P, Wyers M, Gounon P, Walker F (1995). Isolation and partial characterisation of a new strain of Ebola virus.. The Lancet.

[11] Ndayimirije N, Kindhauser MK (2005). Marburg Hemorrhagic Fever in Angola – Fighting Fear and a Lethal Pathogen.. N Engl J Med.

[12] Colebunders R, Tshomba A, Van Kerkhove Maria D, Bausch Daniel G, Campbell P (2007). Marburg Hemorrhagic Fever in Durba and Watsa, Democratic Republic of the Congo: Clinical Documentation, Features of Illness, and Treatment.. The Journal of Infectious Diseases.

[13] Khan Alia S, Tshioko FK, Heymann David L, Le Guenno B, Nabeth P (1999). The Reemergence of Ebola Hemorrhagic Fever, Democratic Republic of the Congo, 1995.. The Journal of Infectious Diseases.

[14] Nakazibwe C (2007). Marburg fever outbreak leads scientists to suspected disease reservoir.. Bulletin of the World Health Organization.

[15] Okware SI, Omaswa FG, Zaramba S, Opio A, Lutwama JJ (2002). An outbreak of Ebola in Uganda.. Tropical Medicine & International Health.

[16] Towner JS, Sealy TK, Khristova ML, Albario CG, Conlan S (2008). Newly Discovered Ebola Virus Associated with Hemorrhagic Fever Outbreak in Uganda.. PLoS Pathog.

[17] Towner JS, Amman BR, Sealy TK, Carroll SAR, Comer JA (2009). Isolation of Genetically Diverse Marburg Viruses from Egyptian Fruit Bats.. PLoS Pathog.

[18] Broussard SR, Comuzzie AG, Leighton KL, Leland MM, Whitehead EM (1997). Characterization of new simian foamy viruses from African nonhuman primates.. Virology.

[19] Makuwa M, Souquiere S, Bourry O, Rouquet P, Telfer P (2007). Complete-genome analysis of hepatitis B virus from wild-born chimpanzees in central Africa demonstrates a strain-specific geographical cluster.. J Gen Virol.

[20] Roy S, Vandenberghe LH, Kryazhimskiy S, Grant R, Calcedo R (2009). Isolation and Characterization of Adenoviruses Persistently Shed from the Gastrointestinal Tract of Non-Human Primates.. PLoS Pathog.

[21] Rich SM, Leendertz FH, Xu G, LeBreton M, Djoko CF (2009). The origin of malignant malaria.. Proceedings of the National Academy of Sciences.

[22] Goldberg TL, Gillespie TR, Rwego IB, Wheeler E, Estoff EL (2007). Patterns of gastrointestinal bacterial exchange between chimpanzees and humans involved in research and tourism in western Uganda.. Biological Conservation.

[23] Rwego IB, Isabirye-Basuta G, Gillespie TR, Goldberg TL (2008). Gastrointestinal Bacterial Transmission among Humans, Mountain Gorillas, and Livestock in Bwindi Impenetrable National Park, Uganda.. Conservation Biology.

[24] Williams JM, Lonsdorf EV, Wilson ML, Schumacher-Stankey J, Goodall J (2008). Causes of death in the Kasekela chimpanzees of Gombe National Park, Tanzania.. American Journal of Primatology.

[25] Boesch C (2008). Why do chimpanzees die in the forest? The challenges of understanding and controlling for wild ape health.. American Journal of Primatology.

[26] Palacios G, Lowenstine L, Cranfield M, Gilardi K, Spelman L (2011). Human metapneumovirus infection in wild mountain gorillas, Rwanda.. Emerging Infectious Diseases.

[27] Sugiyama Y (2004). Demographic parameters and life history of chimpanzees at Bossou, Guinea.. American Journal of Physical Anthropology.

[28] Hanamura S, Kiyono M, Lukasik-Braum M, Mlengeya T, Fujimoto M (2008). Chimpanzee deaths at Mahale caused by a flu-like disease.. Primates.

[29] Werikhe S, Macfie L, Rosen N, Miller P (1997). Can The Mountain Gorilla Survive? Population and Habitat Viability Assessment for *Gorilla gorilla beringei*.

[30] World Health Organization (WHO) T, United Nations Children's Fund (UNICEF) T (2009). Immunization summary: A statistical reference containing data through 2007..

[31] World Health Organization T (2009). World Health Statistics 2009..

[32] Boivin G, De Serres G, Cote S, Gilca R, Abed Y (2003). Human metapneumovirus infections in hospitalized children.. Emerging Infectious Disease.

[33] Weber MW, Mulholland EK, Greenwood BM (1998). Respiratory syncytial virus infection in tropical and developing countries.. Tropical Medicine & International Health.

[34] Arevshatian L, Clements CJ, Lwanga SK, Misore AO, Ndumbe P (2007). An evaluation of infant immunization in Africa: is a transformation in progress?. Bulletin of the World Health Organization.

[35] Dabbagh A, Gacic-Dobo M, Wolfson L, Featherstone D, Strebel P (2008). Progress in Global Measles Control and Mortality Reduction, 2000–2006.. JAMA.

[36] Luna LucianoÂ KleberÂ deÂ S, Panning M, Grywna K, Pfefferle S, Drosten C (2007). Spectrum of Viruses and Atypical Bacteria in Intercontinental Air Travelers with Symptoms of Acute Respiratory Infection.. The Journal of Infectious Diseases.

[37] Glezen WP (2004). The Changing Epidemiology of Respiratory Syncytial Virus and Influenza: Impetus for New Control Measures.. The Pediatric Infectious Disease Journal.

[38] Katz SL (2006). Polio–New challenges in 2006.. Journal of Clinical Virology.

[39] Burgess DC, Burgess MA, Leask J (2006). The MMR vaccination and autism controversy in United Kingdom 1998–2005: Inevitable community outrage or a failure of risk communication?. Vaccine.

[40] Guerrera W, Sleeman JM, Jasper SB, Pace LB, Ichinose TY (2003). Medical Survey of the Local Human Population to Determine Possible Health Risks to the Mountain Gorillas of Bwindi Impenetrable Forest National Park, Uganda.. International Journal of Primatology.

[41] Hartter J, Southworth J (2009). Dwindling resources and fragmentation of landscapes around parks: wetlands and forest patches around Kibale National Park, Uganda.. Landscape Ecology.

[42] Hartter J, Ryan S, Southworth J, Chapman C (2011). Landscapes as continuous entities: forest disturbance and recovery in the Albertine Rift landscape.. Landscape Ecology.

[43] Hartter J, Ryan SJ (2010). Top-down or bottom-up?: Decentralization, natural resource management, and usufruct rights in the forests and wetlands of western Uganda.. Land Use Policy.

[44] Kuehl HS, Nzeingui C, Yeno SLD, Huijbregts B, Boesch C (2009). Discriminating between village and commercial hunting of apes.. Biological Conservation.

[45] Homsy J (1999). Ape Tourism and Human Diseases: How Close Should We Get?.

[46] Cranfield M, Minnis R (2007). An integrated health approach to the conservation of Mountain gorillas *Gorilla beringei beringei*.. International Zoo Yearbook.

[47] Muehlenbein M, Martinez L, Lemke A, Ambu L, Nathan S (2008). Perceived Vaccination Status in Ecotourists and Risks of Anthropozoonoses.. EcoHealth.

[48] Sandbrook C, Semple S (2006). The rules and the reality of mountain gorilla Gorilla beringei beringei tracking: how close do tourists get?. Oryx.

[49] Nakamura M, Nishida T (2009). Chimpanzee Tourism in Relation to the Viewing Regulations at the Mahale Mountains National Park, Tanzania.. Primate Conservation.

[50] Hastings BE, Kenny D, Lowenstine LJ, Foster JW (2001). Mountain gorillas and measles: ontogeny of a wildlife vaccination program.. Proceedings of the American Association of Zoological Veterinarians.

[51] Graczyk T, Mudakikwa A, Cranfield M, Eilenberger U (2001). Hyperkeratotic mange caused by *Sarcoptes scabiei* (Acariformes: Sarcoptidae) in juvenile human-habituated mountain gorillas (*Gorilla gorilla beringei*).. Parasitology Research.

[52] Lusebrink J, Schildgen V, Schildgen O (2009). Novel therapies for an old virus: treatment of RSV infections in the 21st Century.. Expert Review of Anti-Infective Therapy.

[53] Olszewska W, Openshaw P (2009). Emerging drugs for respiratory syncytial virus infection.. Expert Opinion on Emerging Drugs.

[54] Hayden FG (2006). Antivirals for influenza: Historical perspectives and lessons learned.. Antiviral Research.

[55] Whittier AC, Nutter FB, Stoskopf MK (2001). Effects of Hand Rearing on the Reproductive Success of Western Lowland Gorillas in North America; 2001; Brookfield, IL..

[56] Brochier B, Kieny MP, Costy F, Coppens P, Bauduin B (1991). Large-scale eradication of rabies using recombinant vaccinia-rabies vaccine.. Nature.

[57] Sleeman JM, Cameron K, Mudakikwa AB, Nizeyi J-B, Anderson S (2000). Field Anesthesia of Free-Living Mountain Gorillas (*Gorilla gorilla beringei*) from the Virunga Volcano Region, Central Africa.. Journal of Zoo and Wildlife Medicine.

[58] Hosaka K (1995). Epidemics and wild chimpanzee study groups. Mahale: A single flu epidemic killed at least 11 chimpanzees.. Pan African News.

[59] Matsuzawa T, Humle T, Koops K, Biro D, Hayashi M (2004). Wild chimpanzees at Bossou-Nimba: Deaths through a flu-like epidemic in 2003 and the green-corridor project.. Primate Research.

[60] Matsuzawa T (2006). Bossou 30 years.. Pan African News.

[61] Infuso A, Falzon D (2006). European survey of BCG vaccination policies and surveillance in children, 2005.. Eurosurveillance.

[62] Vaccines and Preventable Diseases (2009). Centers for Disease Control and Prevention.. http://www.cdc.gov/vaccines/vpd-vac/default.htm.

[63] Meyer G, Deplanche M, Schelcher F (2008). Human and bovine respiratory syncytial virus vaccine research and development.. Comparative Immunology, Microbiology and Infectious Diseases.

[64] Herfst S, Fouchier RA (2008). Vaccination approaches to combat human metapneumovirus lower respiratory tract infections.. Journal of Clinical Virology.

[65] Jones SM, Feldmann H, Stroher U, Geisbert JB, Fernando L (2005). Live attenuated recombinant vaccine protects nonhuman primates against Ebola and Marburg viruses.. Nature Medicine.

[66] Sullivan NJ, Geisbert TW, Geisbert JB, Xu L, Yang Z-y (2003). Accelerated vaccination for Ebola virus haemorrhagic fever in non-human primates.. Nature.

[67] Warfield KL, Bosio CM, Welcher BC, Deal EM, Mohamadzadeh M (2003). Ebola virus-like particles protect from lethal Ebola virus infection.. Proceedings of the National Academy of Sciences of the United States of America.

[68] Bejon P, Lusingu J, Olotu A, Leach A, Lievens M (2008). Efficacy of RTS,S/AS01E Vaccine against Malaria in Children 5 to 17 Months of Age.. N Engl J Med.

[69] Lell B, Agnandji S, von Glasenapp I, Haertle S, Oyakhiromen S (2009). A Randomized Trial Assessing the Safety and Immunogenicity of AS01 and AS02 Adjuvanted RTS,S Malaria Vaccine Candidates in Children in Gabon.. PLoS ONE.

[70] Gallo R (2009). Toward an HIV preventive vaccine: problems and prospects.. Retrovirology.

[71] Daniel PW, Jeremy F, Sarah R-J (2009). Progress towards a dengue vaccine.. The Lancet Infectious Diseases.

